# Hypoadiponectinemia: A Link between Visceral Obesity and Metabolic Syndrome

**DOI:** 10.1155/2012/175245

**Published:** 2011-10-16

**Authors:** Tiziana Di Chiara, Christiano Argano, Salvatore Corrao, Rosario Scaglione, Giuseppe Licata

**Affiliations:** ^1^Dipartimento Biomedico di Medicina Interna e Specialistica, Università Degli Studi di Palermo, Piazza delle Cliniche 2, 90127 Palermo, Italy; ^2^Divisione di Medicina Interna, Fondazione Istituto San Raffaele Giglio, Contrada Pietra Pollastra, 90015 Cefalù, Palermo, Italy

## Abstract

Metabolic syndrome (MetS) represents a combination of cardiometabolic risk factors, including visceral obesity, glucose intolerance or type 2 diabetes, elevated triglycerides, reduced HDL cholesterol, and hypertension. MetS is rapidly increasing in prevalence worldwide as a consequence of the “epidemic” obesity, with a considerable impact on the global incidence of cardiovascular disease and type 2 diabetes. At present, there is a growing interest on the role of visceral fat accumulation in the occurrence of MetS. In this review, the effects of adipocytokines and other proinflammatory factors produced by fat accumulation on the occurrence of the MetS have been also emphasized. Accordingly, the “hypoadiponectinemia” has been proposed as the most interesting new hypothesis to explain the pathophysiology of MetS.

## 1. Introduction

The association of cardiometabolic risk factors known to date as “metabolic syndrome (MetS)” was described in the past years by several Authors. It has been called also “Syndrome X” or “Deadly quartet visceral fat syndrome” to attribute to the clustering (arterial hypertension and metabolic risk factors) the role of a clinical entity related to insulinresistance [1–3]. There is growing interest in MetS, and the clustering of visceral obesity, diabetes mellitus, hypertriglyceridemia, low levels of high-density lipoprotein-cholesterol (HDL-C), and hypertension has been proposed in several classifications (World Health Organization (WHO) 1999, European Group for the Study of Insulin Resistance (EGIR) 1999, National Cholesterol Education Program's Adult Treatment Panel III (NCEP/ATPIII] 2001), International Diabetes Federation (IDF) 2006) [4–7] (Tables 1, 2, and 3). 

Although insulin resistance has been considered a key factor for the occurrence of MetS, the precise mechanism by which these common metabolic and circulatory disorders cluster in one individual and also why this pathophysiological state is so atherogenic have not been fully clarified. Clinical studies on the morbidities of obesity suggest that the extent of fat accumulation is not necessarily a determinant of development of obesity-related diseases but that body fat distribution is a more important factor for morbidity. Using computed tomography scanning for the analysis of adipose tissues, it has been clarified that visceral adipose tissue accumulation may have a major role in the occurrence of hypertension, diabetes mellitus, hyperlipidemia, and atherosclerotic diseases [8]. Visceral fat accumulation even in mildly obese subjects has been shown to correlate with the occurrence of coronary artery disease, especially in some countries where the prevalence of massive obesity is much lower than in western countries [9]. 

These data suggest that there is an increase in the risk of chronic disease associated with a progressive increase in total adiposity. This hypothesis is supported by several studies following the discovery of Leptin in 1994, indicating that the adipose tissue cannot be considered as an organ that passively stores excess energy but it is an endocrine organ directly involved in the pathophysiology of the MetS and obesity-related cardiovascular disease [10]. Accordingly, a key role in the development of MetS has been recently attributed to visceral obesity inducing hypoadiponectinemia [9, 11, 12].

In this paper we have reported some recent findings to support the hypothesis that hypoadiponectinemia has to be considered a link among visceral obesity and MetS.

## 2. The Adipokines

**Figure 1:**
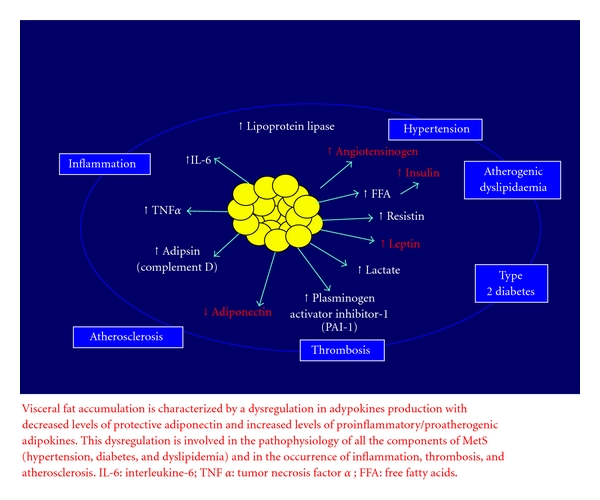
Adverse cardiometabolic effects of products of adipocytes (from Scaglione et al. ([12] modified)).

Adipose tissue participates in the regulation of a variety of homeostatic processes as an endocrine organ that secretes many biologically active molecules such as leptin, tumor necrosis factor-*α* (TNF-*α*), and plasminogen-activator inhibitor type 1 (PAI-1), which contribute to the development of cardiovascular disease [9, 13]. Furthermore, some of these molecules, such as leptin, PAI-1, and adiponectin, are known to contribute to the development of hypertension [9, 13, 14] ( Figure 1).

The molecular mechanism of visceral fat-related disease has been investigated by analysis of the gene-expression profile in both visceral and subcutaneous adipose tissue. Of the gene group classified by function and localization, approximately 20% of all genes in the subcutaneous adipose tissue encode secretory proteins and about 30% in the visceral adipose tissue. These bioactive substances are classified as “adipokines or adipocytokines” and are subdivided into *adipokines adipose tissue specific bioactive substances* (i.e., leptin and adiponectin) and *adipokines abundantly secreted from adipose tissue* which are nonspecific for adipose tissue (PAI-1, TNF-*α*, interleukins (IL), etc) [15]. Adipokines and adipocytokines have a role both in the regulation of the glucose and lipid metabolism, in the control of oxidative stress, and in the maintenance of the vascular wall integrity (Figure 1) [12, 14, 15].

## 3. Adiponectin

Adiponectin was discovered during gene-expression profiling of human adipose tissue conducted by the human cDNA project. Located on chromosome 3q27, a locus for diabetes susceptibility [16], adiponectin encodes a secretory protein expressed exclusively in adipose tissue. Adiponectin contains 244 amino acids, a signal peptide, a collagen-like domain at its N-terminus, and a globular domain at its C-terminus, which shares sequence similarities with collagens X and VIII as well as complement factor C1q. Despite the absence of primary sequence similarity, the crystal structure of the C-terminal globular domain resembles that of TNF-*α*. During the same period, two other groups identified ACRP30 and AdipoQ as mouse homologs of adiponectin. Adiponectin has anti-inflammatory and antiatherogenic properties [14, 15]. The adiponectin receptors (adipoR1 and adipoR2) have been cloned. AdipoR1 is expressed ubiquitously, whereas adipoR2 is predominantly expressed in the liver, and both are correlated positively with insulin sensitivity. The plasma range of adiponectin in humans is 3–30 *μ*g/ml, accounting for 0.01% of total plasma protein [16]. Adiponectin exists in a wide range of multimer complexes in plasma and combines via its collagen domain to create three major oligomeric forms: trimers, hexamers, and a high-molecular-mass form. A smaller form of adiponectin that includes the globular domain cleaves proteolytically from full-length adiponectin and exists in plasma, although in very small amounts [16, 17].

### 3.1. The Biological Effects of Adiponectin in Experimental Models

Data from animal models demonstrate the biological effects of adiponectin. In particular, it is able to influence insulin sensitivity, glucose, and lipid metabolism and to modulate blood pressure regulation and hypertensive target organ disease [15, 16]. 

In addition, cell biological studies demonstrate multiple antiatherogenic functions for adiponectin. When the endothelial barrier is injured by attacking factors (oxidized LDL, chemical substances, and mechanical stress), adiponectin accumulates in the subendothelial space of vascular walls by binding to subendothelial collagen, at which point antiatherogenic properties of adiponectin become apparent. In addition, low levels of adiponectin associated with obesity, MetS, and diabetes favor T-lymphocyte recruitment and contribute to adaptive immune response during atherogenesis in a mouse model of atherosclerosis [18]. Adiponectin also attenuates growth-factor-induced proliferation of visceral smooth muscle cells by the inhibition of mitogen-activated protein kinase. It suppresses foam-cell formation by the inhibition of the expression of scavenger receptors [19] and protects plaque from rupture by inhibition of matrix metalloproteinase function through the induction of IL-10-dependent production of tissue inhibitor metalloproteinase. In addition, adiponectin-deficient mice show enhanced left ventricular hypertrophy and increased mortality under pressure overload. On the contrary, adenovirus mediated adiponectin supplementation attenuates cardiac hypertrophy in response to pressure overload [19].

### 3.2. The Biological Effects of Adiponectin in Humans

The first indication that adiponectin might have a role in human obesity derives from report of Hu et al., indicating that the expression of adiponectin using northern blots is reduced in the adipose tissue of obese mice and humans [20, 21]. However, data on adiponectin in humans are increased by the introduction of an adiponectin immunoassay [22]. Accordingly, plasma adiponectin levels are found higher in women than in men and in nonobese than in obese subjects [12].

Therefore, adiponectin is the only fat protein that has downregulation in relation to weight gain, and it is possible that an accumulation of visceral fat might produce inhibiting factors for adiponectin synthesis or secretion, such as TNF-*α* [23, 24].

Lower plasma levels of adiponectin are also predictive of type 2 diabetes mellitus (DM) and are found in diabetic subjects and in patients with hypertriglyceridemia, low HDL-C, and hypertension [15, 16, 25]. Human subjects with more cardiovascular risk factors, or with MetS, would be expected to have lower plasma adiponectin levels [26]. 

In this area of interest, conflicting evidences have emerged about the prognostic role of adiponectin on cardiovascular disease (CVD). In fact, antiatherogenic effects of adiponectin have also demonstrated in some clinical studies, indicating that higher adiponectin levels are associated with a reduced risk of acute myocardial infarction in men [27]. Subjects with hypoadiponectinemia (plasma levels <4 *μ*g/mL) have an increased risk of coronary heart disease and multiple metabolic risk factors [28]. Subjects with renal insufficiency and with higher adiponectin levels are free from cardiovascular death for a longer period than those with renal insufficiency and low adiponectin levels [29]. These data are also confirmed by the results of some large epidemiological studies [30, 31]. 

On the contrary, other prospective studies [32, 33] do not report a significant cardioprotective effect of adiponectin. These conflicting results raise the possibility that adiponectin may have different prognostic implications in population at different risk of vascular disease. 

In addition to the studies linking plasma adiponectin levels to various human diseases, human genetic studies provide evidence of an association between lower adiponectin levels and obesity, DM, dyslipidemia, hypertension, MetS, insulin resistance, and coronary artery disease (CAD) [15, 18]. Interestingly, the association of the adiponectin genetic variation with obesity, MetS, and DM has been recently reported in a Taiwanese elderly population, suggesting the genetic effects of adiponectin inherited at birth could be extended all the way to this later stage of life [34]. These data suggest that in addition to hypoadiponectinemia associated with visceral fat accumulation, genetic hypoadiponectinemia may exhibit a clinical phenotype of MetS.

### 3.3. Relationship among Adiponectin, Obesity, and Insulin Resistance

Adiponectin may be considered as the molecular link between obesity and insulin resistance. Is hypoadiponectinemia the cause or the result of obesity and adipose tissue-specific insulin resistance in humans?

Animal experiments using injection of recombinant adiponectin proteins and the adiponectin knockout (KO) mice clearly demonstrate that adiponectin produces effects on both body weight and insulin sensitivity in the liver and muscle [18]. However, the severity of obesity observed in adiponectin deficiency is totally outweighed by that in leptin-deficient mice (*ob/ob*), suggesting that leptin is the master hormone of long-term weight regulation in animals. Therefore, hypoadiponectinemia is not likely the main cause of obesity and adipose tissue-specific insulin resistance. Low adiponectin expression, on the other hand, is found in many animal models of obesity [34].

The metabolic effects of adiponectin, including lowering glucose, enhancing fatty acid *β*-oxidation, and improving insulin sensitivity, appear to be convincing and significant. Therefore, it is reasonable to propose that hypoadiponectinemia is the result of obesity and adipose tissue-specific insulin resistance, but it is the mediator from obesity to the insulin resistance in the other peripheral tissues (such as liver and muscle) and associated metabolic outcomes. Human genetic studies clearly demonstrate that adiponectin gene variants are one of the causes of obesity and insulin resistance, usually with an odds ratio of less than 2, which is as expected for a polygenic disorder. Hypoadiponectinemia also does not predict obesity in a human prospective study while it is shown to predict the development of type 2 DM. Interestingly, significant body weight reduction in humans was shown to raise plasma adiponectin levels accompanied with improved insulin sensitivity [35].

The next question then is what does cause adiponectin expression in obesity, and how does it happen? It is possible to hypothesize that the expression of adiponectin in the adipose tissue is inhibited by the mechanisms related to obesity-induced insulin resistance, such as inflammation. This inhibition could be reversed by weight reduction, which improves adipose tissue-specific insulin sensitivity. In human subjects treated with PPAR*γ*2 agonist, the insulin sensitizer that mainly acts in the adipose tissue increases plasma adiponectin, by approximately twofold in spite of a significant body weight gain, which is almost routinely seen in this kind of treatment [36]. These findings indicated that improving adipose-specific insulin sensitivity is able to increase adiponectin gene expression irrespective of the changes in adiposity. 

Therefore, adipose tissue-specific insulin sensitivity rather than general adiposity itself determines the adiponectin expression in adipose tissues. Secondary to the increased plasma adiponectin, the whole body insulin sensitivity would be expected to improve. 

The molecular mechanisms of obesity-induced adipose tissue-specific insulin resistance may be elucidated by studying the molecular regulation of adiponectin gene expression.

### 3.4. Relationship among Adiponectin, Hypertension, and Insulin Resistance

Furuhashi et al. [37] reported that only hypertensive patients with insulin resistance showed a decreased adiponectin concentration. However, the cause-effect relationship among hypoadiponectinemia, insulin resistance, and hypertension has not been clearly elucidated. Even though the consensus has been that insulin resistance is correlated with hypertension, [38] the association between insulin and hypertension is controversial [39]. In fact, homeostasis model assessment (HOMA) index was not significantly different between hypertensive and normotensive subjects in some studies, but, as a specific finding, plasma adiponectin level significantly decreased with an increase in blood pressure, even in the normotensives without insulin resistance or diabetes [13, 40, 41]. These results indicate that hypoadiponectinemia may affect the pathogenesis of hypertension at a very early stage without involving insulin resistance. In addition, Lindsay et al. [42] reported that there were loci on chromosomes 2, 3, 9, and 10 affecting the circulating adiponectin concentration in the Pima Indian population, suggesting the possibility of an unknown modulator of adiponectin level. However, further data are required to support this hypothesis. There are 4 possible reasons for the negative correlation between hypertension and plasma adiponectin concentration. First, plasma adiponectin concentration was independently correlated with the vasodilator response to reactive hyperemia, so adiponectin concentration could be an independent parameter of endothelial function. Endothelial dysfunction is an important feature of the early stage of atherosclerosis, which is related to pathogenic conditions including hypertension [43]. Furthermore, in adiponectin-KO mice, hypoadiponectinemia causes diet-induced hypertension. Second, an increase in sympathetic nerve activity, which is common in hypertensives, may inhibit adiponectin gene expression via alfa-adrenergic stimulation [44]. Third, the reciprocal association of adiponectin and high-sensitive C-reactive protein or increased risk of arteriosclerosis suggests that a low adiponectin concentration might enhance the predisposition to hypertension via vascular injury [13]. Fourth, activation of the renin-angiotensin system may be induced in adipose tissue by hypoadiponectinemia, resulting in an increase in fat mass and blood pressure [45].

## 4. Conclusions

**Figure 2:**
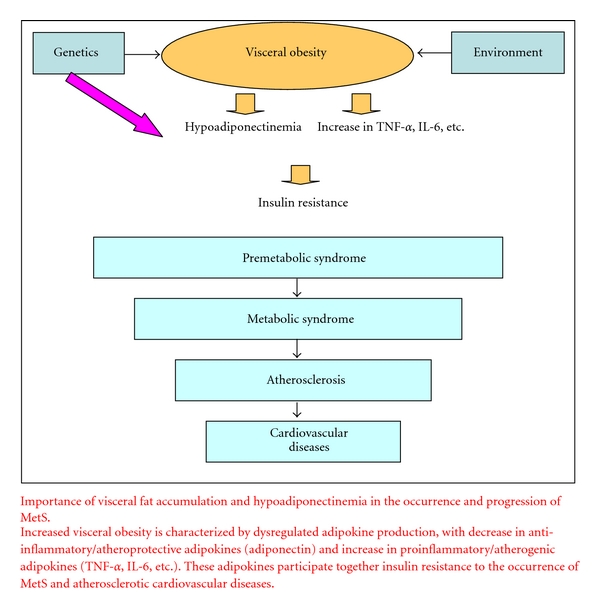
Progression and outcomes of visceral obesity.

Although risks that cluster in MetS are common, it is likely that the cluster occurs coincidentally. Adiponectin is a unique and essential adipocytokine that is produced very abundantly in adipocytes and stably present in the plasma at very high concentration. In healthy subjects, adiponectin carries out its roles for preventing development of vascular changes and the impairment of glucose and lipid metabolism, which may be induced by a variety of attacking factors, such as chemical substances, mechanical stress, or nutritional loading, like a firefighter who is putting out small fires to keep them from becoming big. Studies on the genetic mutation of adiponectin gene in human subjects and the KO mice clearly demonstrate that adiponectin may play a key role also in the prevention of MetS [8, 17, 24, 46]. Acquired hypoadiponectinemia observed in obesity, especially with visceral fat accumulation, and hypertension, is much more frequent than genetic hypoadiponectinemia. Hypoadiponectinemia together with the increase of other adipocytokines (i.e., TNF-alfa or PAI-1) induced by the accumulation of visceral obesity might be a major background of vascular changes as well as metabolic disorders, including insulin resistance, which are the characteristics of so-called “metabolic syndrome” (Figure 2).

##  Conflict of Interests

All the authors declare that there is no conflict of interest.

## Figures and Tables

**Table 1:** Principal metabolic syndrome definitions.

	WHO (1999)	EGIR (1999)	NCEP ATP III (2001)
Fasting plasma glucose	Glucose intolerance, IGT or diabetes, and/or insulin resistance together with two or more of the following:	Insulin resistance (defined as hyperinsulinemia—top 25% or fasting insulin values among the nondiabetic population). Plus two of the following:	3 or more of the following factors:
		≥6.1 mmol/L (110 mg/dl) but nondiabetic	5.6 mmol/L (100 mg/dl)

Blood pressure	>140/90 mmHg	≥140/90 mmHg or treatment	≥130/≥ 85 mmHg

Triglycerides	Raised plasma triglycerides ≥1.7 mmol/L (150 mg/dl) and/or	(i) 2.0 mmol/L (178 mg/dl) or treatment and/or	1.7 mmol/L (150 mg/dl)

HDL-cholesterol	Men: <0.9 mmol/L (35 mg/dl) Women: <1.0 mmol/L (39 mg/dl)	<1.0 mmol/L (39 mg/dl) or treatment	Men: <1.03 mmol/L (40 mg/dl) Women: <1.29 mmol/L (50 mg/dl)

Obesity	Men: waist-hip ratio >0.90 Women: waist-hip ratio >0.85 and/or BMI >30 Kg/^2^	Men: waist circumference ≥ 94 cm Women: waist circumference ≥80 cm	Men: waist circumference >102 cm Women waist circumference >88 cm

Microalbuminuria	Urinary albumin excretion rate ≥ 20 *μ*g/min or albumin: creatinine ratio ≥30 mg/g		

WHO:World Health Organization; EGIR: European Group for the Study of Insulin Resistance; NCEP/ATPIII: National Cholesterol Education Program's Adult Treatment Panel III.

**Table 2:** International Diabetes Federation (IDF) metabolic syndrome worldwide definition (IDF, 2006).

Central obesity	Waist circumference—ethnicity specific plus any two of the following:
Raised triglycerides	≥1.7 mmol/L (150 mg/dl) or specific treatment

Reduced HDL-C	<1.03 mmol/L (40 mg/dl in males) <1.29 mmol/L (50 mg/dl) in females or specific treatment

Raised blood pressure	Systolic ≥130 mmHg or Diastolic ≥85 mmHg or treatment of previously diagnosed hypertension

Raised fasting plasma glucose	Fasting plasma glucose ≥5.6 mmol/L (100 mg/dl) or previously diagnosed Type 2 diabetes. If >5.6 mmol/L or 100 mg/dl, oral glucose tolerance test is strongly recommended but is not necessary to define presence of the syndrome.

This is the first classification indicating central obesity as an obligatory component of MetS.

HDL-C: High-density lipoprotein-cholesterol.

**Table 3:** Values of waist circumference recommended by IDF criteria, according to ethnicity.

	Male	Female
Europids	>94 cm	>80 cm
South Asians	>90 cm	>80 cm
Chinese	>90 cm	>80 cm
Japanese	>85 cm	>90 cm
South and Central Americans	Use south Asian recommendations	
South Saharian Africans	Use European data	
Eastern Mediterraneum and Middle East	Use European data	

A major issue for the IDF consensus consultation was the fact that criteria used for central obesity in Asian and other populations could be different from those used in the West.

IDF: International Diabetes Federation.

## References

[1] Avogaro P, Crepaldi G (1965). Essential hyperlipidemia, obesity and diabetes. *Diabetologia*.

[2] Reaven GM (1988). Banting lecture: role of insulin resistance in human disease. *Diabetes*.

[3] Kaplan NM (1989). The deadly quartet: upper-body obesity, glucose intolerance, hypertriglyceridemia, and hypertension. *Archives of Internal Medicine*.

[4] (1999). Definition and classification of diabetes mellitus and its complications. *Report of a WHO document. WHO/NCD/NCS/99.2*.

[5] Balkau B, Charles MA (1999). Comment on the provisional report from the WHO consultation. European Group for the Study of Insulin Resistance (EGIR). *Diabetic Medicine*.

[6] Expert Panel on Detection, Evaluation, and Treatment of High Blood Cholesterol in Adults (2001). Executive summary of the third report of the National Cholesterol Education Program (NCEP) expert panel on detection, evaluation, and treatment of high blood cholesterol in adults (adult treatment panel III). *Journal of the American Medical Association*.

[7] Alberti KGMM, Zimmet P, Shaw J (2006). Metabolic syndrome : a world-wide definition: a consensus statement of the International Diabetes Federation. *Diabetic Medicine*.

[8] Matsuzawa Y, Funahashi T, Kihara S, Shimomura I (2004). Adiponectin and metabolic syndrome. *Arteriosclerosis, Thrombosis, and Vascular Biology*.

[9] Nakamura T, Tokunaga K, Shimomura I (1994). Contribution of visceral fat accumulation to the development of coronary artery disease in non-obese men. *Atherosclerosis*.

[10] Bergman RN, Kim SP, Hsu IR (2007). Abdominal obesity: role in the pathophysiology of metabolic disease and cardiovascular risk. *American Journal of Medicine*.

[11] Weyer C, Funahashi T, Tanaka S (2001). Hypoadiponectinemia in obesity and type 2 diabetes: close association with insulin resistance and hyperinsulinemia. *Journal of Clinical Endocrinology and Metabolism*.

[12] Scaglione R, Di Chiara T, Cariello T, Licata G (2010). Visceral obesity and metabolic syndrome: two faces of the same medal?. *Internal and Emergency Medicine*.

[13] Iwashima Y, Katsuya T, Ishikawa K (2004). Hypoadiponectinemia is an independent risk factor for hypertension. *Hypertension*.

[14] Zhang H, Cui J, Zhang C (2010). Emerging role of adipokines as mediators in atherosclerosis. *World Journal of Cardiology*.

[15] Matsuzawa Y (2006). The metabolic syndrome and adipocytokines. *FEBS Letters*.

[16] Kadowaki T, Yamauchi T (2005). Adiponectin and adiponectin receptors. *Endocrine Reviews*.

[17] Takahashi M, Arita Y, Yamagata K (2000). Genomic structure and mutations in adipose-specific gene, adiponectin. *International Journal of Obesity*.

[18] Okamoto Y, Folco EJ, Minami M (2008). Adiponectin inhibits the production of CXC receptor 3 chemokine ligands in macrophages and reduces T-lymphocyte recruitment in atherogenesis. *Circulation Research*.

[19] Shibata R, Ouchi N, Ito M (2004). Adiponectin-mediated modulation of hypertrophic signals in the heart. *Nature Medicine*.

[20] Tarquini R, Lazzeri C, Laffi G, Gensini GF (2007). Adiponectin and the cardiovascular system: from risk to disease. *Internal and Emergency Medicine*.

[21] Hu E, Liang P, Spiegelman BM (1996). AdipoQ is a novel adipose-specific gene dysregulated in obesity. *Journal of Biological Chemistry*.

[22] Arita Y, Kihara S, Ouchi N (1999). Paradoxical decrease of an adipose-specific protein, adiponectin, in obesity. *Biochemical and Biophysical Research Communications*.

[23] Halleux CM, Takahashi M, Delporte ML (2001). Secretion of adiponectin and regulation of apM1 gene expression in human visceral adipose tissue. *Biochemical and Biophysical Research Communications*.

[24] Maeda N, Takahashi M, Funahashi T (2001). PPAR*γ* ligands increase expression and plasma concentrations of adiponectin, an adipose-derived protein. *Diabetes*.

[25] Díez JJ, Iglesias P (2003). The role of the novel adipocyte-derived hormone adiponectin in human disease. *European Journal of Endocrinology*.

[26] Shargorodsky M, Boaz M, Goldberg Y (2009). Adiponectin and vascular properties in obese patients: is it a novel biomarker of early atherosclerosis. *International Journal of Obesity*.

[27] Pischon T, Girman CJ, Hotamisligil GS, Rifai N, Hu FB, Rimm EB (2004). Plasma adiponectin levels and risk of myocardial infarction in men. *Journal of the American Medical Association*.

[28] Kumada M, Kihara S, Sumitsuji S (2003). Association of hypoadiponectinemia with coronary artery disease in men. *Arteriosclerosis, Thrombosis, and Vascular Biology*.

[29] Zoccali C, Mallamaci F, Tripepi G (2002). Adiponectin, metabolic risk factors, and cardiovascular events among patients with end-stage renal disease. *Journal of the American Society of Nephrology*.

[30] Koenig W, Khuseyinova N, Baumert J, Meisinger C, Löwel H (2006). Serum concentrations of adiponectin and risk of type 2 diabetes mellitus and coronary heart disease in apparently healthy middle-aged men. results from the 18-Year follow-up of a large cohort from Southern Germany. *Journal of the American College of Cardiology*.

[31] Frystyk J, Berne C, Berglund L, Jensevik K, Flyvbjerg A, Zethelius B (2007). Serum adiponectin is a predictor of coronary heart disease: a population-based 10-year follow-up study in elderly men. *Journal of Clinical Endocrinology and Metabolism*.

[32] Sattar N, Wannamethee G, Sarwar N (2006). Adiponectin and coronary heart disease: a prospective study and meta-analysis. *Circulation*.

[33] Lawlor DA, Smith GD, Ebrahim S, Thompson C, Sattar N (2005). Plasma adiponectin levels are associated with insulin resistance, but do not predict future risk of coronary heart disease in women. *Journal of Clinical Endocrinology and Metabolism*.

[34] Vozarova B, Stefan N, Lindsay RS (2002). Low plasma adiponectin concentrations do not predict weight gain in humans. *Diabetes*.

[35] Duncan BB, Schmidt MI, Pankow JS (2004). Adiponectin and the development of type 2 diabetes: the atherosclerosis risk in communities study. *Diabetes*.

[36] Yang WS, Jeng CY, Wu TJ (2002). Synthetic peroxisome proliferator-activated receptor-*γ* agonist, rosiglitazone, increases plasma levels of adiponectin in type 2 diabetic patients. *Diabetes Care*.

[37] Furuhashi M, Ura N, Higashiura K (2003). Blockade of the renin-angiotensin system increases adiponectin concentrations in patients with essential hypertension. *Hypertension*.

[38] Ferrannini E, Natali A, Capaldo B, Lehtovirta M, Jacob S, Yki-Järvinen H (1997). Insulin resistance, hyperinsulinemia, and blood pressure: role of age and obesity. *Hypertension*.

[39] Haffner SM (1993). Editorial: insulin and blood pressure: fact or fantasy?. *Journal of Clinical Endocrinology and Metabolism*.

[40] Avery PJ, Patel SK, Ibrahim IM, Walker M, Keavney BD Common variation in the adiponectin gene has an effect on systolic blood pressure.

[41] Ohashi K, Ouchi N, Matsuzawa Y (2011). Adiponectin and hypertension. *American Journal of Hypertension*.

[42] Lindsay RS, Funahashi T, Krakoff J (2003). Genome-wide linkage analysis of serum adiponectin in the Pima Indian population. *Diabetes*.

[43] Vita JA, Keaney JF (2002). Endothelial function: a barometer for cardiovascular risk?. *Circulation*.

[44] Fasshauer M, Klein J, Neumann S, Eszlinger M, Paschke R (2001). Adiponectin gene expression is inhibited by *β*-adrenergic stimulation via protein kinase A in 3T3-L1 adipocytes. *FEBS Letters*.

[45] Jones BH, Standridge MK, Taylor JW, Moustaïd N (1997). Angiotensinogen gene expression in adipose tissue: analysis of obese models and hormonal and nutritional control. *American Journal of Physiology*.

[46] Hiuge-Shimizu A, Maeda N, Hirata A (2011). Dynamic changes of adiponectin and S100A8 levels by the selective peroxisome proliferator-activated receptor-*γ* agonist rivoglitazone. *Arteriosclerosis, Thrombosis, and Vascular Biology*.

